# Knowledge of and attitudes towards cardiopulmonary resuscitation among junior doctors and medical students in Upper Egypt: cross-sectional study

**DOI:** 10.1186/s12245-020-00277-x

**Published:** 2020-04-22

**Authors:** Zeinab Mohammed, Ahmed Arafa, Yaseen Saleh, Mohamed Dardir, Asmaa Taha, Hassnaa Shaban, Eman Mohammed AbdelSalam, Jon Mark Hirshon

**Affiliations:** 1 Department of Public Health and Community Medicine, Faculty of Medicine, Beni-Suef University, Beni-Suef, Egypt; 2 College of Medicine, University of Illinois at Chicago, 1853 West Polk Street, 112 CMW, Chicago, IL 60612 USA; 3 Faculty of Medicine, Beni-Suef University, Beni-Suef, Egypt; 4 Department of Surgery, Faculty of Medicine, Beni-Suef University, Beni-Suef, Egypt; 5 Department of Emergency Medicine, School of Medicine, University of Maryland, Baltimore, USA

**Keywords:** Medical students, Junior doctors, Cardiopulmonary resuscitation, Knowledge, Training, Attitude, LMIC, Global medicine, Emergency medicine

## Abstract

**Background:**

Cardiopulmonary resuscitation (CPR) is a fundamental skill that should be acquired by all medical community members. This study aims to evaluate the knowledge and attitudes of junior doctors and medical students towards CPR and CPR training at Beni-Suef University Hospital in Upper Egypt, a representative region with conditions common to LMIC settings.

**Participants and methods:**

In this cross-sectional study, a total of 205 participants (60 junior doctors and 145 medical students) responded to a self-administered questionnaire assessing their knowledge regarding basic life support (BLS) and CPR techniques in neonates, children, and adults, in addition to attitudes towards the importance and necessity of CPR and CPR training.

**Results:**

Of the 60 junior doctors that participated in the study, only 31.7% had adequate knowledge of CPR, but up to 95% reported positive attitudes towards CPR training. Among the 145 medical student participants, only 6.2% had adequate knowledge of CPR, while 91% reported positive attitudes towards training. Deficiencies in CPR knowledge were more apparent in questions related to CPR in children and neonates. Junior doctors and medical students with previous CPR training demonstrated significantly better CPR knowledge than their counterparts without prior training. A statistically significant positive correlation was detected between CPR knowledge and attitude towards CPR training among medical students (*r* = 0.41, *p* < 0.001).

**Conclusion:**

The results of this study demonstrate suboptimal and inadequate CPR knowledge among junior doctors and medical students in a representative hospital in Upper Egypt. However, participants reported overwhelmingly positive attitudes and eagerness towards the implementation of CPR training. Further research needs to be done to establish CPR skill proficiency as well as to investigate barriers to CPR training, effectiveness of available programs, and the potential implementation of such a program in Egypt and other LMICs.

## Introduction

Cardiopulmonary resuscitation (CPR) is a critical, life-saving skill for healthcare professionals (HCPs) in emergency departments (EDs) and other health care settings. Although the clinical outcome of CPR depends on multiple factors, such as the initial condition of the patient and the duration of cardiac arrest, performing high-quality CPR significantly improves patient outcomes [1]. Presentation to the ED with cardiac arrest is most commonly seen among adults; however, it can also occur and is particularly challenging for HCPs to manage in children and neonates. Accordingly, comprehensive CPR is a critical skill for HCPs employed in the ED [2]. International standards recommend CPR as a requirement for graduation from medical school and junior doctors should be proficient enough to perform CPR from their first day of practice [3]. Furthermore, the inclusion of CPR early in medical curricula is thought to increase medical students’ awareness and appreciation of this vital skill [2]. Despite being well-established and one of the most effective resuscitative measures for patients in cardiac arrest, lack of knowledge, insufficient training, and inadequate practice of CPR have been documented among medical students and junior doctors in high-income as well as low- and middle-income countries (LMICs) [4–8].

In Egypt, CPR certification is not required for graduation from the medical school nor for registration in the Egyptian Medical Syndicate following graduation. Therefore, it can be hypothesized that both medical students and junior doctors in Egypt have inadequate knowledge of CPR and have neutral or negative attitudes towards the practice. This study aims to assess the knowledge and attitudes of junior doctors and medical students at Beni-Suef University’s Hospital and College of Medicine respectively towards CPR and CPR training.

## Participants and methods

### Study design and setting

This cross-sectional study was conducted in Beni-Suef University’s College of Medicine and the Beni-Suef University Hospital in Upper Egypt from January to the end of March 2017. Beni-Suef University Hospital is one of the largest hospitals in Upper Egypt that offers secondary and tertiary healthcare to more than 3 million residents in Beni-Suef Governorate. This hospital serves a mixture of patients (in terms of urbanized/rural ratios, income, and educational attainment) that is representative of most Upper Egyptian governorates.

### Sampling

We used Epi-Info version 7 StatCalc from the Center for Disease Control and Prevention (CDC), to calculate the sample size. The following criteria were set: the inadequate knowledge rate at 50%, the confidence level at 90%, and the margin of error at 5%. Systematic random sampling was used to select medical students from the rosters of the 4th, 5th, and 6th academic years, while all junior doctors in internal medicine and the surgical departments were contacted to participate in this study.

### Ethical considerations

The study proposal was approved by the Research Ethics Committee of Beni-Suef University, and participants were informed of the details and aims of the study prior to consenting to participation.

### Data collection tool

Modifying previous literature, we created a semi-structured questionnaire assessing adequate knowledge of CPR (based on the American Heart Association 2015 guidelines) in addition to attitudes towards CPR. The knowledge section contained 20 multiple-choice items that were previously used and validated in similar studies [4, 9] while the attitude section included seven items also adapted from a previous study [10]. Knowledge questions were scored as binary variables; correct answers were given 1 point, while incorrect answers and “do not know” responses were given 0 points. Thus, the total knowledge score could potentially range between 0 and 20 points. Attitude questions were awarded 2 points for a positive response, 1 point for a neutral response, and 0 points for a negative response. Accordingly, attitude scores could range from 0 to 14 points. Participants were asked to answer the questionnaire without consulting any materials or textbooks and to answer according to their current, best knowledge. Questionnaires returned empty were excluded from the study.

### Statistical analyses

Data were analyzed using the software Statistical Package for Social Science (SPSS Inc. Released 2009, PASW Statistics for Windows, version 18.0: SPSS Inc., Chicago, Illinois, USA). Those who scored > 50% (10/20) of the total knowledge points were considered having adequate knowledge, while those who scored > 50% (7/14) of the total attitude score were considered to have a positive attitude. A chi-square statistical test was used to detect the association between previous CPR training and current knowledge as well as attitude. A Spearman’s correlation test was used to detect the association between knowledge and attitude.

## Results

The response rate to this study was 44.2% among junior doctors and 39% among medical students. Of the 60 participating junior doctors (40 males and 20 females), 29 were house officers (first year graduates) and 31 were new residents (after completing the house officer’s year). The 145 participating medical students (33 males and 112 females) were distributed among the academic years as follows: 85 were in their 4th year, 46 in their 5th year, and 16 in their 6th (final) year. Thus, it should be noted the majority of participating medical students were still in their early, primarily preclinical years of education.

### Knowledge

**Fig. 1:**
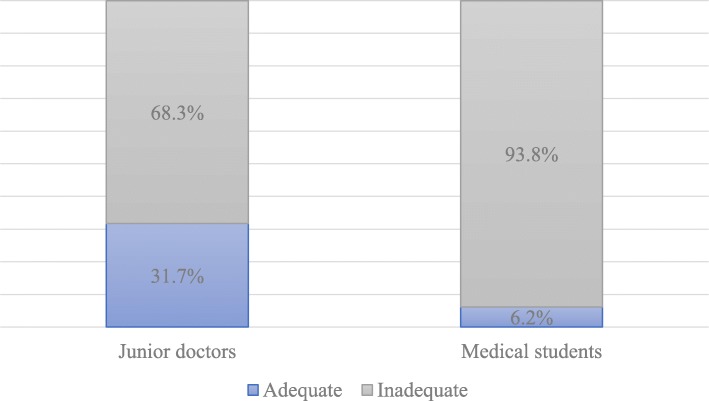
Assessment of junior doctors and medical students’ knowledge of CPR (*p* < 0.001)

More than two thirds of the participating junior doctors (68.3%) had inadequate knowledge of CPR, scoring less than 50% on the questionnaire, while the vast majority of medical students (93.8%) also showed inadequate knowledge. The deficiencies were most apparent for both groups in the questions assessing children- and neonate-specific CPR knowledge. For example, only 26.7% of junior doctors and 26.9% of medical students were able to identify the location of chest compressions in infants. Even lower rates of knowledge were particularly noticeable for questions covering the depth of compressions in children and in neonates as well as questions on rescue breathing in infants. Specifically, less than 20% of both junior doctors and medical students could correctly identify the depth of compressions in children and neonates, with strikingly as low as 4.8% of medical students able to answer correctly with respect to neonates. Both groups struggled (< 25%) with the topic of rescue breathing in infants, with the junior doctors (23.3%) performing close to twice as well as the medical students (12.4%). Most junior doctors (93.3%) and half of medical students (55.2%), however, could at least recognize the BLS acronym. Generally, junior doctors had better knowledge than medical students, scoring significantly better on average (*p* < 0.001) (Table 1, Fig. 1).

**Table 1:** Correct responses to questions assessing CPR knowledge among junior doctors and medical students

Question/Knowledge area assessed	Junior doctors # (%)	Medical students # (%)
BLS abbreviation	56 (93.3)	80 (55.2)
Response to cardiac arrest on the road	25 (41.7)	55 (37.9)
Location of chest compressions in adults	27 (45.0)	56 (38.6)
Location of chest compressions in infants	16 (26.7)	39 (26.9)
Rescue breathing in infants	14 (23.3)	18 (12.4)
Depth of compressions in adults	31 (51.7)	49 (33.8)
Depth of compressions in children	7 (11.7)	28 (19.3)
Depth of compressions in neonates	12 (20.0)	7 (4.8)
Rate of chest compressions in adults and children	16 (26.7)	36 (24.8)
Current updated order of CPR intervention	11 (18.3)	11 (7.6)
Recommended universal compressions to ventilation ratio	34 (56.7)	40 (27.6)
CPR attempted inside a hospital rather than ambulatory	47 (78.3)	97 (66.9)
Window of effectiveness for CPR from onset of arrest	22 (36.7)	26 (17.9)
Artificial respirations > CPR in respiratory arrest	39 (65.0)	63 (43.4)
Most people who receive CPR survive	19 (31.7)	13 (9.0)
Reversible and irreversible brain damage	34 (56.7)	58 (40.0)
Blood flow cessation for > 10 h and cell death	25 (41.7)	46 (31.7)
CPR optimum period	39 (65.0)	66 (45.5)
Ambulatory compression-only CPR	18 (30.0)	19 (13.1)
Survival rate after defibrillation	35 (58.3)	59 (40.7)

### Attitude

**Fig. 2:**
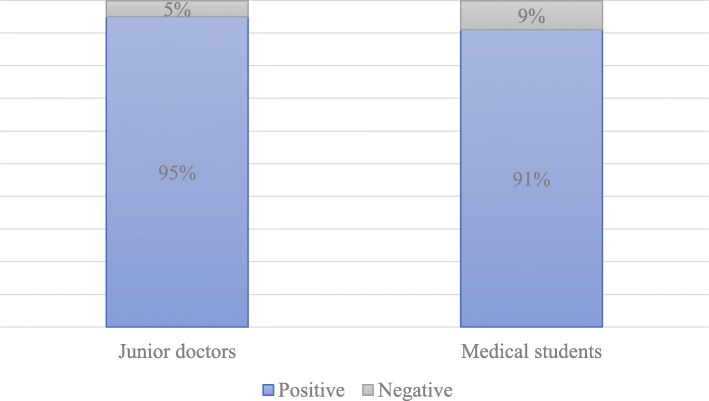
Assessment of junior doctors and medical students’ attitudes towards CPR (*p* = 0.256)

Despite gaps in knowledge, both junior doctors and medical students held overwhelmingly positive attitudes towards CPR training, with no statistical difference between them (95% in junior doctors and 91% in students; *p* = 0.256, Fig. 2). The majority of junior doctors and medical students agreed there is a necessity for CPR training (95.0% and 61.1%, respectively) and that it should be added to the curriculum (83.3% and 77.9%, respectively). However, less than a third of junior doctors (31.7%) and less than an eighth of medical students (11.0%) believed that their university could offer this training (Table 2). Reasons for the discrepancies between the junior doctors and medical students are unknown but may be due to a lack of clinical experience in the medical student group.

**Table 2:** Reported positive attitudes towards CPR training among medical students and junior doctors

Question	Junior doctors # (%)	Medical students # (%)
BLS training is necessary	57 (95.0)	88 (61.1)
BLS workshop	46 (76.7)	118 (81.4)
BLS should be part of curriculum	50 (83.3)	113 (77.9)
Undergraduates should do CPR whenever possible	47 (78.3)	125 (86.2)
If I knew CPR well, I wouldn’t hesitate to perform CPR	50 (83.3)	98 (67.6)
I would like to transfer my CPR knowledge to colleagues	48 (80.0)	116 (80.0)
I think my university is able to provide CPR training	19 (31.7)	16 (11.0)

### Previous training

As expected, junior doctors who reported previous CPR training had significantly better CPR knowledge that those without previous training (odds ratio (OR) 3.6, 95% CI 1.1–11.8, *p* = 0.029). Likewise, trained medical students showed better CPR knowledge that those without training (OR 4.6, 95% CI 1.1–18.5, *p* = 0.043). There was no statistical significance in the difference of attitudes between the two groups with previous CPR training and the respective groups without training, albeit those with training generally tended to have a more positive score (Table 3).

**Table 3:** Association between attending previous CPR training and current knowledge or attitudes

Groups		Training # (%)	No training # (%)	*p* value	OR (95% CI)
Knowledge					
Junior doctors	Adequate	14 (43.8)	5 (17.9)	0.029	3.6 (1.1–11.8)
	Inadequate	18 (56.3)	23 (82.1)		
Medical students	Adequate	4 (16.7)	5 (4.2)	0.043	4.6 (1.1–18.5)
	Inadequate	20 (83.3)	114 (95.8)		
Attitude					
Junior doctors	Positive	32 (100.0)	25 (89.3)	0.096	---
	Negative	0 (0.0)	3 (10.7)		
Medical students	Positive	23 (95.8)	107 (89.9)	0.320	2.6 (0.3–20.8)
	Negative	1 (4.2)	12 (10.1)		

### Knowledge-attitude correlation

**Fig. 3:**
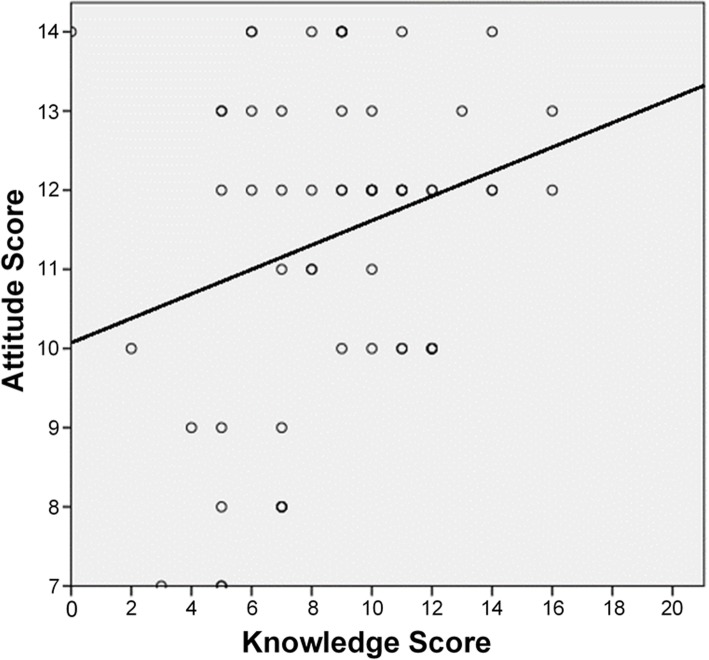
Correlation between knowledge score and attitude score among junior doctors (*r* = 0.17, *p* = 0.193)

**Fig. 4:**
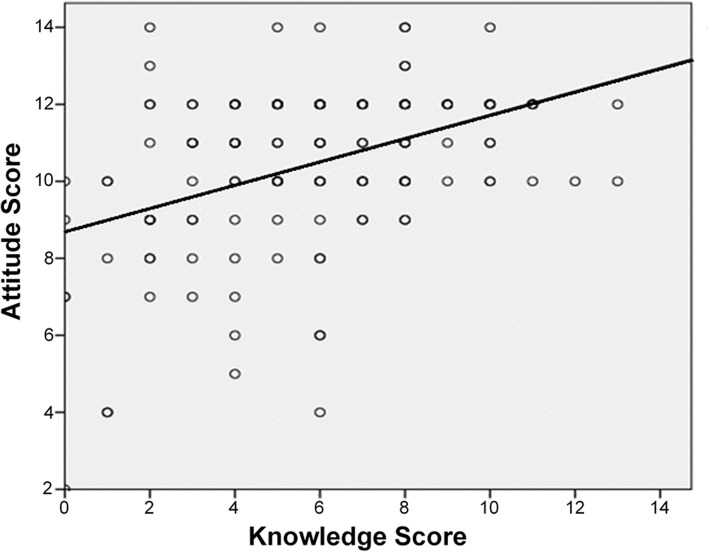
Correlation between knowledge score and attitude score among medical students (*r* = 0.41, *p* < 0.001)

A slight positive trend (*r* = 0.17) was initially seen when analyzing the correlation between CPR knowledge scores and attitudes towards CPR training among junior doctors; however, this was found to be statistically insignificant (*p* = 0.193) (Fig. 3). In contrast, a relatively strong, positive correlation between knowledge and attitude was found to be statistically significant among medical students (*r* = 0.41, *p* < 0.001) (Fig. 4).

## Discussion

Cardiac arrest is a common presentation in the emergency department, especially in tertiary care hospitals. Accordingly, HCPs employed in these hospitals should have mastered first aid and BLS procedures. While CPR training is obligatory for employment in healthcare fields in many countries, there is no such requirement in Egypt. Unsurprisingly, our study indicates a stark lack of proficiency in CPR among junior doctors and medical students in a representative tertiary care hospital in Upper Egypt. In spite of this lack of knowledge and thus potential proficiency, attitudes towards CPR were generally positive.

Similar knowledge deficiencies were reported among doctors and medical students in other LMICs, including South India [11], Sri Lanka [8], Jamaica [7], Nigeria [12], and Ethiopia [10]. Inadequate knowledge of CPR in such LMICs may be attributable to the lack of continuous and formal training. This is supported by our finding that junior doctors and medical students with previous formal, structured CPR training were shown to have better CPR knowledge (and thus retention). In accordance with this, a previous study from Pakistan similarly stressed the need for continuous and regular training to improve CPR knowledge among medical students [13].

Nevertheless, it should be noted that efficient CPR training may be difficult to implement in an institution lacking prior infrastructure, requiring specific and potentially costly materials and qualified tutors [14]. Restrictions due to cost can be especially problematic in underprivileged institutions with large student bodies (and high student/teacher ratios). A previous study detected significant disparities in CPR training partially attributable to financial reasons, with lower income areas in the United States lacking in bystander CPR training [15]. In line with this, Upper Egyptian governorates like Beni-Suef are the least privileged governorates in Egypt in terms of average household income and educational attainment [16]. Such financial factors may represent barriers that prevent junior doctors and medical students with relatively low incomes from receiving CPR training independently [15]. However, as this is speculative, further research regarding the barriers to CPR training in Egypt is needed.

Our study also revealed a positive correlation among medical students between knowledge of CPR and their attitudes towards CPR training. This is of particular importance considering medical students are still in the primarily educational phase of their career paths. Establishing increased knowledge and positive attitudes towards CPR increases the likelihood of students retaining this information and wanting to implement it in the future. Accordingly, it is imperative to make CPR training attractive and accessible to such student populations. Such improvements in basic CPR knowledge could be attained in numerous ways including simple lectures, seminars, and brochures.

Despite the deficiencies in knowledge detected, it must be noted that the participants reported overwhelmingly positive attitudes towards CPR training in addition to their readiness to spread the knowledge they would acquire to peers. This willingness could facilitate both the development of future training sessions and the inclusion of CPR education within medical curricula as both the students are eager to learn, and the potential teachers are keen to teach.

Nonetheless, some limitations of this study should be considered. First, this study reported a relatively low response rate making the results vulnerable to skewing factors including non-response bias. Second, the study evaluated CPR knowledge only via multiple-choice selection. Therefore, it is difficult to draw conclusions about functional CPR practice within the hospital as there was no assessment of practical skill and proficiency in resuscitation. Lastly, especially with resource-limited settings, we should be careful to generalize beyond the setting of the study. Beni-Suef University Hospital is an academic tertiary care hospital that accurately represents conditions seen across Upper Egypt but may not be as characteristic of more urbanized and higher income areas found in Lower Egypt such as in Cairo.

## Conclusion

In conclusion, this study revealed suboptimal CPR knowledge among junior doctors and medical students in Beni-Suef, Upper Egypt. However, despite these deficiencies, we demonstrated a positive attitude and eagerness towards the implementation of CPR training. Future research should focus on the barriers to attain CPR training and the effectiveness of the available CPR training programs, as well as potential implementation of such programs in a setting like Beni-Suef University’s College of Medicine.

## Data Availability

The data that were generated and analyzed in this study are mostly included within the published article. However, source material and the raw datasets are available from the corresponding author upon request.

## References

[1] Steen PA, Kramer-Johansen J (2008). Improving cardiopulmonary resuscitation quality to ensure survival. Curr Opin Crit Care.

[2] Zaheer H, Haque Z (2009). Awareness about BLS (CPR) among medical students: status and requirements. J Pak Med Assoc.

[3] Morgan R, Westmoreland C (2002). Survey of junior hospital doctors’ attitudes to cardiopulmonary resuscitation. Postgrad Med J.

[4] Almesned A (2014). Basic life support knowledge of healthcare students and professionals in the Qassim University. Int J health Sci.

[5] Wang J (2015). Performance of cardiopulmonary resuscitation during prolonged basic life support in military medical university students: a manikin study. World J Emerg Med.

[6] Kumari KM (2014). Clinical awareness of do’s and don’ts of cardiopulmonary resuscitation (CPR) among university medical students-a questionnaire study. J Clin Diagn Res.

[7] Howell P (2014). Physicians’ knowledge of cardiopulmonary resuscitation guidelines and current certification status at the University Hospital of the West Indies, Jamaica. West Indian Med J.

[8] Ralapanawa, D.M.P.U.K., et al., A study on the knowledge and attitudes on advanced life support among medical students and medical officers in a tertiary care hospital in Sri Lanka*.* BMC Res notes, 2016. 9(1): p. 462-462. 10.1186/s13104-016-2270-5 PMC5059911 27729072

[9] Chandrasekaran S (2010). Awareness of basic life support among medical, dental, nursing students and doctors. Indian J Anaesth.

[10] Tsegaye W, Tesfaye M, Alemu M. Knowledge, attitude and practice of cardiopulmonary resuscitation and associated factors in Ethiopian university medical students. J Gen Pract. 2015;3:206.

[11] Aroor AR (2014). Awareness about basic life support and emergency medical services and its associated factors among students in a tertiary care hospital in South India. J Emerg Trauma Shock.

[12] Olajumoke TO (2012). Cardiopulmonary resuscitation - knowledge, attitude & practices In Osun State, Nigeria. J West Afr Coll Surg.

[13] Abbas A, Bukhari SI, Ahmad F (2011). Knowledge of first aid and basic life support amongst medical students: a comparison between trained and un-trained students. J Pak Med Assoc.

[14] Sasson C (2013). Barriers and facilitators to learning and performing cardiopulmonary resuscitation in neighborhoods with low bystander cardiopulmonary resuscitation prevalence and high rates of cardiac arrest in Columbus, OH. Circ Cardiovasc Qual Outcomes.

[15] Blewer AL (2017). Cardiopulmonary resuscitation training disparities in the United States. J Am Heart Assoc.

[16] Ministry of investment and international cooperation, E., Beni-Suef governorate. 2019: https://www.investinegypt.gov.eg/english//pages/geography.aspx?GovernorateId=5. Accessed Dec. 2019.

